# Public Trust in Dentists among Arabic Populations

**DOI:** 10.1155/2023/8359780

**Published:** 2023-02-24

**Authors:** Aceil Al-Khatib, Radwan Abed Alhaleem, Rama Qaffaf

**Affiliations:** ^1^Department of Oral Medicine and Surgery, Faculty of Dentistry, Jordan University of Science and Technology, P.O. Box 3030, Irbid 22110, Jordan; ^2^Jordanian Ministry of Health, Previously, Faculty of Dentistry, Jordan University of Science and Technology, P.O. Box 3030, Irbid 22110, Jordan

## Abstract

**Objective:**

To investigate public trust in dentists, fear of dentists, factors related to trust, and the impact of the COVID-19 pandemic on the trust in dentists.

**Materials and Methods:**

We used an Arabic online anonymous survey to collect data from a random population of 838 adults to investigate public trust in dentists, the factors they perceive to affect trust, their perception of key factors in the dentist-patient relationship, fear of dentists, and the impact of the COVID-19 pandemic on the level of their trust in the dentists.

**Results:**

Eight hundred thirty-eight subjects with a mean age of 28.5 responded to the survey (595 (71%) females, 235 (28%) males, and 8 (1%) did not specify their gender). More than half trust their dentist. The COVID-19 pandemic did not decrease trust in dentists according to 62.2%. There were significant gender differences in reporting fear of dentists (*p* < 0.001) and in the perception of factors affecting trust (*p*=0.028). Honesty was chosen by 583 (69.6%), competence by 549 (65.5), and dentist's reputation by 443 (52.9%).

**Conclusions:**

The findings of this study show that the majority of the public trust dentists, more females reported fear of dentists, and the majority perceived honesty, competence, and reputation as key factors affecting trust in the dentist-patient relationship. The majority reported that the COVID-19 pandemic did not have a negative impact on their trust in dentists.

## 1. Introduction

Trust in dentists is a key pillar of the dentist-patient relationship. Without trust, patients would not seek dental treatment or visit dentists to seek dental care nor would they reveal the details of their illness or submit to treatment [1]. The result would be aggravated dental problems and deterioration of patients' dental and oral health, which would likely cause unnecessary pain and suffering. The outcome of not seeking dental care would likely impact the patient's health and could affect the quality of their life and predispose them to medical complications. In spite of the importance of patients' trust in dentists and the role it plays in the dentist-patient relationship, it has not been adequately investigated, at least in Arabic regions or Arabic speaking populations. Furthermore, although the public health message, which has focused on the dental clinic as a high risk-aerosol-inducing environment, may have affected the public trust in dentists, the impact of coronavirus disease (COVID-19) pandemic on patients' trust in dentists is not well understood despite the fact that it is now known to most populations that the novel virus that causes COVID-19, known as severe acute respiratory syndrome coronavirus 2 (SARS-CoV-2), is transmitted by droplets and aerosols that are highly infectious and produced during most dental procedures. A recent study found that 19% of Italians reported that they have little trust in their dentists' infection control practices during the pandemic, and 48% of the study population reported extreme trust in their dentists and their sanitization and sterilization procedures [2].

Though many miscellaneous attempts in the literature clearly define the meaning and interpretation of the term “Trust,” Hall et al. [1] defined the term simply as “the optimistic acceptance of a vulnerable situation” in which the truster believes the trustee will care for the truster's interests. Studies that investigated trust in the patient-physician context showed that trust is a keystone in the physician-patient relationship as gaining patients' trust is a fundamental trait of a good clinician [3]. Trust is influenced by the clinician's technical competency [3, 4] and their communication skills [5, 6]. However, as a result of trusting the physician, patients were more likely to adhere to the treatment provided to them by their respective physician [5]. It was also provided that a patient's doubt in their dentist can provoke dental anxiety owing to the fear of the unknown as patients are reluctant to trust a dentist that is unwilling to answer their questions, thereby stressing the importance of trust in the dentist-patient relationship [7]. The issue of trust (or lack thereof) has gained more interest in recent publications, and a certain trend of mistrust in physicians is emerging as reported by recent studies from China and India whose authors point to a worldwide mistrust in healthcare [8, 9], although according to a previous study, a decline in patients' trust in their physicians became salient in the 1990s [10]. Although rare, reports about trust and mistrust in dentists indicate that mistrusting the dentist's ability can aggravate patients' fear and anxiety, especially when the patient does not comprehend what the dentist is doing in their mouth [7].

Research on trust in dentists has recently garnered attention for its importance in promoting oral health and as a key factor in the dentist-patient relationship [11]. Despite the importance of trust, there was, until recently, little research and guidance to dentists on how to build trust or how to be trustworthy [12]. One of the previous studies on trust in dentists was carried out in Australia; it showed that the majority of the adult population had trust in their dentists [12, 13].

COVID-19 is a new source of concern and fear for both dentists and patients [14]. This recent challenge may also strain the patient-dentist relationship and could have an everlasting impact on patients' trust in dentists. This impact is not fully understood, not only because the virus that causes COVID-19 is novel but because the concept of trust in dentists is not fully investigated and understood as we have mentioned previously. Furthermore, although the public health message which has focused on the dental clinic as a high risk-aerosol-inducing environment may have affected the public trust in dentists, the gap in knowledge about trust in dentists in the COVID-19 era is worthy of investigation.

Therefore, the objectives of this study were as follows: (1) to investigate subjects' trust in dentists and fear of dentists, (2) identify which trust-related factors are perceived to be important by the public, and (3) explore if COVID-19 had any impact on public trust in dentists.

### 1.1. Study Population and Methodology

An anonymous online Arabic questionnaire was created by the authors using a Google Form. The questionnaire was used to evaluate the public's trust in dentists and whether the COVID-19 pandemic had any impact on the trust in dentists. The questionnaire was divided into four sections. The first section was a description of the purpose of the study and an informed consent. Invitees were asked to fill in the questionnaire if they would consent to participate. The second section included questions about demographic characteristics of participants. The third section included questions investigating how much respondents agreed with statements about trust in dentists following The Dentist Trust Scale [13] and questions to investigate the association between trust and fear. The fourth section included a question on whether the COVID-19 pandemic had any impact on subjects' trust in dentists and questions on the factors that affect trust in dentists and which factor was perceived as the factor most affecting trust in dentists. The study was approved by the Institutional Review Board (IRB) of King Abdullah University Hospital (KAUH)-Irbid-Jordan (Ref. 85/136/2020). The questionnaire was validated and piloted before inviting subjects to participate. Then, the Google Form questionnaire's link was posted on Facebook (group and personal accounts) and sent via WhatsApp. To minimize selection bias by the authors, we asked potential respondents to share the questionnaire's link with their families and friends and those who live in other countries.

### 1.2. Statistical Analysis

Collected data were analysed by Microsoft Office Excel 2013 (Microsoft Corporation, Redmond, Washington) and by the statistical program for social sciences IBM SPSS Statistics version 26 (IBM SPSS Statistics for Windows, Version 26.0. IBM Corp., Armonk, NY).

Descriptive statistics were used to analyze the data. To examine differences between genders, Pearson's chi-square test or Fisher's exact test were used. A *p* value less than 0.05 was considered statistically significant.

## 2. Results

From December 11, 2020 to February 4, 2021, eight hundred thirty-eight subjects responded to the survey (595 (71%) females, 235 (28%) males, and 8 (1%) did not specify their gender). Seven hundred forty-five (89%) were from Jordan, and 11% were from other countries.  Table 1 shows the demographic characteristics of the respondents. Answering all questions on demographics was not required to ensure voluntariness and eliminate untrue answers.

Table 2 shows the responses to questions about subjects' agreement with statements indicating their perception of important factors of the dentist-patient relationship. 44.7% (*n* = 375) agreed and 22.6% (*n* = 189) strongly agreed that they had no worries about putting their oral health in the hands of the dentist. The majority (46.1% (*n* = 386) and 23.6% (*n* = 198)) agreed and strongly agreed that they completely trust dentists' decisions about which dental treatments are best. More than half (53%) agreed or strongly agreed with the statement “in general, you completely trust your dentist,” 29.8% were neutral, and 16.4% reported that they disagreed or strongly disagreed with this statement. There was a statistically significant association between the responses to “in general, you completely trust dentists” and “do you fear dentists” *X*^2^ (12, *N* = 838) = 37.9, *p* < 0.001.

**Figure 1:**
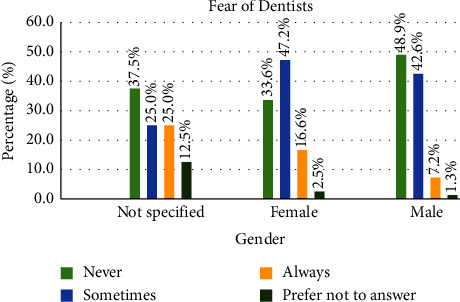
The percentage of responses to “do you fear dentists” by gender.

Regarding the pandemic impact on subjects' trust in dentists, Figure 1 shows the percentage of responses to “do you fear dentists” by gender.

Regarding the perception of subjects about the factors affecting their trust in dentists, subjects could choose more than one factor: honesty was chosen by 583 (69.6%), competence was perceived to be an important factor by 549 (65.5), and dentist's reputation was an important factor according to 443 (52.9%). The cost of treatment was reported to be important by 255 (30.4%).

**Figure 2:**
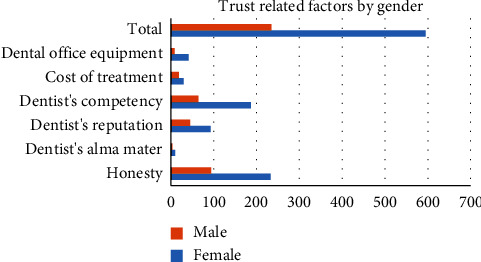
The most important factor that can affect trust as perceived by gender. 8 respondents did not specify their gender.

Figure 2 shows the most important factor that can affect trust as perceived by gender. There were statistically significant differences between gender and age groups in responding to a question about the most important factor affecting trust [*X*^2^ (10, *N* = 838) = 20, *p*=0.028; *X*^2^ (25, *N* = 838) = 49, *p*=0.003], respectively. Honesty was perceived to be the most important factor, followed by competency, dentist's reputation, and only 14 (1.7%) reported that the university the dentist attended (alma mater) was the most important trust factor. On the other hand, there was no significant difference between genders; the majority perceived the dentist's treatment decisions to be in the patient's best interest. 62.2% reported that the COVID-19 pandemic did not affect their trust in dentists, while 18.9% reported that the pandemic negatively impacted their trust in dentists.

## 3. Discussion

Trust is a keystone in the dentist-patient relationship. Building trust between dentists and their patients can be improved by understanding prospective patients' perception of the key factors that might affect trust [15]. It can be theorized that the COVID-19 pandemic and the public's attention to the risks entailed in the exposure to a deadly virus in a dental office may have negatively impacted the public trust in dentists. The results of this investigation indicate that the majority of respondents trust their dentists and that the COVID-19 pandemic did not have a negative impact on their trust in dentists, although a minority reported that the pandemic decreased their trust in dentists. This finding is in line with a recent investigation from Italy [2]. It is evident that the population of the present study believed their dentist would not mislead them and that the treatment decisions made by their dentists are in the patient's best interest. Furthermore, the majority reported having no worry in putting their oral health in the hands of their dentist.

Although 41.7% of the population agreed that “dentists only think about what is best for themselves,” 69.7% reported to completely trust dentists' decisions about which dental treatments are best. These findings suggest that if trust is to be defined by Hall et al. [1] as the belief that the trustee will care for the truster's interests, then the discrepancy may be attributed to the assumption that the patients believe that their dentist's interests align with the patient's own interests. Indeed, a strong argument would support the fact that building trust in the dentist-patient relationship is in the best interest of dentists, patients, and oral health systems [15].

The results of this study show that 17–40% of the respondents' answers to the questions of the modified Dentist's Trust Scale were “neutral” which can be understood to mean that a significant proportion of society is undecided about whether they can trust or distrust dentists. This finding is important and can be used by dentists and dental associations to implement trust building strategies that enhance societal trust in the profession of dentistry.

Although the results of this study show that the majority of our population trust dentists, which is in alignment with the results of a similar study on an Australian population, both studies showed comparable results with means ranging from 3 to 3.9. However, a slight deviation between this study and the Australian study can be spotted when the participants were asked about whether they thought that “dentists think only about what is best for their patients,” in this context, it seems that the Australian population has more trust in their dentists. A plausible explanation can be attributed to methodological and population differences.

In agreement with a previous study [16], the results of this investigation showed significant gender differences regarding fear of dentists, and more females reported to sometimes or always fear dentists. Findings of this study indicate that there might be a possible association between fear and trust. The association between trust in dentists and the fear of dentists warrants further investigation as dental fear could lead to avoidance behavior and deterioration of dental health [17] and can revolve around many issues such as feelings of shame, fear of being judged by the dentist [18], fear of chocking, fear of blood, and lack of trust in the competency of dentist. Overall, good communication practices can mitigate fear of treatment [19] because patients who need to see a dentist are often frightened [20] and some feel uncomfortable expressing their fear of dentists or dental procedures [21].

In terms of the factors affecting trust, both males and females chose honesty (females: 39.2%-males: 40%) and competency (females: 31.4%-males: 27.2%). Curiously, the dentist's alma mater was chosen by a few as the most important factor affecting trust.

Previous and recent research has established that honesty is a fundamental characteristic of the best dentist-patient relationship [22]. Improving ethical practices and building trust in the dentist-patient relationship cannot be achieved without honesty and competency. Although receiving quality dental care by a competent dentist is the goal of each dental patient, misleading patients by dishonest dentists is a violation of the patient's rights and a trust eroding behavior. Consequently, behaviors that erode public trust in dentists would certainly harm the position of the profession of dentistry in society and violate the ethical principles of practicing dentistry [23, 24].

### 3.1. Limitations

Data used in this study were collected by using an online self-administered questionnaire; therefore, the findings from this study are subject to some limitations. The most important limitation is different sources of bias including recall bias and selection bias. The majority of the respondents were females, although we could not determine the response rate, but the results show that most respondents were in the younger age group. This could be due to the fact that a significant percentage of seniors may not have access to smart phones or access to the Internet. Thus, the results may lack external validity; further research with other populations will determine the generalizability of the findings of this study.

## 4. Conclusions

The findings of this study add to the current literature on the trust in dentists. The results suggest an association between fear of dentists and trust in dentists, which should be considered in future research on the dentist-patient relationship. Findings provide evidence that honesty, although perceived to be the most important factor affecting trust, honesty, and competence, go hand in hand and are perceived to be the most important factors affecting trust in dentists among different genders and age groups. This study also highlights the need for further research on the relationship between trust and using dental services. Its findings can be used to raise awareness about the importance of adopting trust building behaviors that enhance the societal perception of dentists and promote oral health and the need to avoid practices that could erode public trust in dentists.

## Figures and Tables

**Table 1:** Demographic characteristics of the respondents.

Variables	*N*	(%)	Total responses
Gender			830
Females	595	71.7	
Males	235	28.3	
Age			836
18–29	503	60.2	
30–39	120	14.4	
40–49	161	19.3	
50–59	48	5.7	
59–69	4	0.5	
Educational level			832
High school	91	10.9	
Community college	53	6.4	
Bachelor degree	566	68	
Postgraduate	122	14.7	
Monthly income			800
<$750	275	34.4	
$750–$1500	321	40.1	
>$1500	204	25.5	

**Table 2:** Answers to a modified (a validated Arabic questionnaire was used) dentist's trust scale.

Statement on a 5-point Likert scale	Strongly disagree *N*/(%)		Disagree *N*/(%)	Neutral *N*/(%)	Agree *N*/(%)		Strongly agree *N*/(%)
A dentist would never mislead you about anything	15/1.8	148/17.7		294/35.1	284/33.9	97/11.6	
You have no worries about putting your oral health in the hands of the dentist	13/1.6	116/13.8		145/17.3	375/44.7	189/22.6	
Dentists do what is best for their patients	12/1.4	79/9.4		242/28.9	361/43.1	144/17.2	
Dentists think only about what is best for their patients	42/5.0	227/27.1		331/39.5	166/19.8	72/8.6	
Dentists are totally honest in telling their patients about all the different treatment options available for their conditions	13/1.6	104/12.4		213/25.4	356/42.5	152/18.1	
You completely trust dentists' decisions about which dental treatments are best	12/1.4	83/9.9		159/19.0	386/46.1	198/23.6	
Dentists think only about what is best for themselves	32/3.8	150/17.9		306/36.5	230/27.4	120/14.3	
Sometimes dentists do not pay full attention to what patients are trying to tell them	49/5.8	229/27.3		276/32.9	209/24.9	75/8.9	
Dentists are extremely thorough and careful	7/0.8	79/9.4		239/28.5	367/43.8	146/17.4	
In general, you trust your dentist	16/1.9	122/14.5		250/29.8	318/37.9	132/15.7	

## Data Availability

The data used to support the findings of this study are available from the corresponding author upon reasonable request.

## References

[1] Hall M. A., Dugan E., Zheng B., Mishra A. K. (2001). Trust in physicians and medical institutions: what is it, can it be measured, and does it matter?. *The Milbank Quarterly*.

[2] Aquilanti L., Gallegati S., Temperini V. (2020). Italian response to coronavirus pandemic in dental care access: the DeCADE study. *International Journal of Environmental Research and Public Health*.

[3] Pellegrini C. (2017). Trust: the keystone of the physician-patient relationship. *Bulletin of the American College of Surgeons*.

[4] Kerasidou A. (2020). Artificial intelligence and the ongoing need for empathy, compassion and trust in healthcare. *Bulletin of the World Health Organization*.

[5] Thom D. H. (2001). Physician behaviors that predict patient trust. *Journal of Family Practice*.

[6] Levinson W., Lesser C. S., Epstein R. M. (2010). Developing physician communication skills for patient-centered care. *Health Affairs*.

[7] Fico A. E., Lagoe C. (2018). Patients’ perspectives of oral healthcare providers’ communication: considering the impact of message source and content. *Health Communication*.

[8] Nie J. B., Li L., Gillett G., Tucker J. D., Kleinman A. (2018). The crisis of patient-physician trust and bioethics: lessons and inspirations from China. *Developing World Bioethics*.

[9] Kane S., Calnan M. (2016). Erosion of trust in the medical profession in India: time for doctors to act. *International Journal of Health Policy and Management*.

[10] Bloche M. G. (2004). Trust and verify. *Health Affairs*.

[11] Tiwari T., Maliq N. N., Rai N. (2022). Evaluating Trust in the Patient–Dentist Relationship: A Mixed-Method Study. *JDR Clinical & Translational Research*.

[12] Armfield J. M., Ketting M., Chrisopoulos S., Baker S. R. (2017). Do people trust dentists? Development of the dentist trust Scale. *Australian Dental Journal*.

[13] Meng L., Hua F., Bian Z. (2020). Coronavirus disease 2019 (COVID-19): emerging and future challenges for dental and oral medicine. *Journal of Dental Research*.

[14] Song Y., Luzzi L., Brennan D. S. (2020). Trust in dentist-patient relationships: mapping the relevant concepts. *European Journal of Oral Sciences*.

[15] Saatchi M., Abtahi M., Mohammadi G., Mirdamadi M., Binandeh E. S. (2015). The prevalence of dental anxiety and fear in patients referred to Isfahan Dental School, Iran. *Dental Research Journal*.

[16] Avramova N. (2022). Dental fear, anxiety, and phobia; causes, diagnostic criteria and the medical and social impact. *Journal of Mind and Medical Sciences*.

[17] Calladine H., Currie C. C., Penlington C. (Apr 2022). A survey of patients’ concerns about visiting the dentist and how dentists can help. *Journal of Oral Rehabilitation*.

[18] Cserző D., Bullock A., Cowpe J., Bartlett S. (2022). Professionalism in the dental practice: perspectives from members of the public, dentists and dental care professionals. *British Dental Journal*.

[19] Bishop M. A. (2018). The patient-dentist relationship and the future of dentistry. *British Dental Journal*.

[20] Armfield J. M., Heaton L. J. (2013). Management of fear and anxiety in the dental clinic: a review. *Australian Dental Journal*.

[21] Ustrell-Torrent J. M., Buxarrais-Estrada M. R., Ustrell-TorrentRiutord-Sbert P. (2021). Ethical relationship in the dentist-patient interaction. *Journal of clinical and experimental dentistry*.

[22] Welie J. V. (2019). Patient autonomy as a necessary but limited ethical principle in shaping the dentist-patient relationship. *The Journal of forensic odonto-stomatology*.

[23] American Dental Association A. D. A. (2018). *Principles of Ethics Code & of Professional Conduct*.

[24] Dyer T. A., Owens J., Robinson P. G. (2016). The acceptability of healthcare: from satisfaction to trust. *Community Dent Health*.

